# Harnessing Nanohybridized Niclosamide for Precision Mpox Therapeutics

**DOI:** 10.1002/adhm.202404818

**Published:** 2025-02-23

**Authors:** N. Sanoj Rejinold, Geun‐woo Jin, Jin‐Ho Choy

**Affiliations:** ^1^ Intelligent Nanohybrid Materials Laboratory (INML) Department of Chemistry College of Science and Technology Dankook University Cheonan 31116 Republic of Korea; ^2^ R&D Center Hyundai Bioscience Co. LTD Seoul 03759 Republic of Korea; ^3^ Division of Natural Sciences The National Academy of Sciences Seoul 06579 Republic of Korea; ^4^ Tokyo Tech World Research Hub Initiative (WRHI) Institute of Innovative Research Institute of Science Tokyo Yokohama 226‐8503 Japan

**Keywords:** bioavailability enhancement, Mpox antiviral therapy, nanohybrid formulations, niclosamide repurposing, poxvirus therapeutic strategies

## Abstract

Niclosamide, initially developed as an anthelmintic, has recently emerged as a potential antiviral, showing efficacy against diverse viral threats, including Mpox. As the global health landscape faces recurrent Mpox outbreaks, repurposing niclosamide through advanced material strategies offers promising therapeutic avenues. This article explores the antiviral mechanisms of niclosamide, focusing on how innovative nano‐hybrid formulations enhance its bioavailability and pharmacological performance. By leveraging nanohybridization, niclosamide's limitations—such as poor solubility and bioavailability—are addressed, enabling targeted delivery and sustained release. Early preclinical studies reveal that niclosamide disrupts Mpox replication and entry processes, suggesting its utility as a therapeutic option against poxvirus infections. Looking forward, further in vitro, animal models, and clinical investigations are essential to optimize its application and dosing for Mpox. With continued development in advanced materials, nanohybrid niclosamide could become a critical tool in managing Mpox and related viral threats, offering an accessible, cost‐effective option for outbreak preparedness.

## Graphical abstract

This study investigates the potential of nanohybridized niclosamide as a therapeutic agent for Mpox, focusing on enhanced bioavailability, improved antiviral efficacy, and controlled drug release achieved through nanoengineering. The research emphasizes significant advancements in formulation strategies, mechanistic insights, and therapeutic outcomes, underscoring its relevance in precision medicine. The findings position nanohybridized niclosamide as a promising antiviral treatment, addressing existing challenges in Mpox therapy and paving the way for future clinical applications.

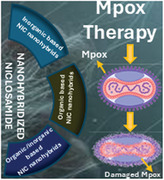

## 1 Introduction

First identified in the Democratic Republic of the Congo in the 1970s, Mpox (formerly known as “monkeypox”) has historically caused sporadic infections and outbreaks. In response to its global spread in 2022 and 2024, the WHO declared Mpox, a Public Health Emergency of International Concern (PHEIC) and adopted the new name to mitigate discrimination and stigma.^[^ ^1^, ^2^, ^3^ ^]^

Mpox is an orthopoxvirus closely related to the variola virus, the causative agent of smallpox characterized by its brick‐shaped virion and a double‐stranded DNA genome. The viral structure includes an outer lipid envelope, which encases the viral core containing the double‐stranded DNA genome. Additionally, the virus contains several key proteins, such as the viral envelope proteins (e.g., A27, B5), which are involved in cell entry and fusion, and the core proteins that play a role in replication and immune evasion. The structural complexity of the Mpox virus makes it a challenging target for antiviral therapies (Figure S1, Supporting Information) (**Table** **1**).^[^ ^4^ ^]^

**Table 1:** Outlining different pox viruses in history, the clinical drugs used for their treatment, and their limitations.

Pox Virus	Disease	Clinical Drugs	FDA‐Approved Drugs	Limitations	Refs.
Variola	Smallpox	Cidofovir, Tecovirimat (ST‐246), Brincidofovir	Tecovirimat (TPOXX)	‐ Tecovirimat: Limited human testing, mostly based on animal studies (FDA approved under the Animal Rule). ‐ Potential drug resistance. ‐ Not effective for prevention.	[5, 6]
Vaccinia	Vaccinia virus infection	Tecovirimat, Cidofovir, Brincidofovir	No specific FDA‐approved treatment (for complications)	‐ No approved treatment for severe complications (e.g., progressive vaccinia). ‐ Immune‐compromised individuals at higher risk.	[6, 7, 8]
Mpox	Mpox	Tecovirimat, Brincidofovir, Cidofovir	Tecovirimat (TPOXX)	‐ Limited clinical data for efficacy in humans. ‐ Availability is limited in some regions. ‐ Not fully evaluated in large human trials.	[6, 7, 8]
Cowpox	Cowpox	Supportive care (antiviral drugs in severe cases)	No specific FDA‐approved drug	‐ No specific antiviral treatment approved by the FDA. ‐ Rare and usually mild, so treatments are not well‐studied.	[9]
Molluscum contagiosum	Molluscum contagiosum	Cidofovir (off‐label), Imiquimod	No specific FDA‐approved antiviral treatment	‐ Treatments are typically physical (cryotherapy, curettage). ‐ Cidofovir: Off‐label, potential toxicity. ‐ Imiquimod: Variable effectiveness.	[10]
Orf	contagious pustular dermatitis	Supportive care, Cidofovir (severe cases)	No specific FDA‐approved antiviral treatment	‐ Disease is self‐limiting, so antivirals are rarely used. ‐ Cidofovir: Limited by nephrotoxicity.	[11, 12]

Mpox is primarily a zoonotic disease, transmitted from animals to humans through contact with infected animals or contaminated materials. Human‐to‐human transmission occurs through direct contact with infectious lesions, bodily fluids, or respiratory droplets, although it is less contagious than smallpox.^[^ ^13^, ^14^ ^]^

Mpox initially presents with flu‐like symptoms, followed by a characteristic rash or pustular lesions.^[^ ^15^, ^16^, ^17^, ^18^, ^19^ ^]^ While traditionally endemic to West and Central Africa, cases were generally sporadic and associated with wildlife contact.

However, in recent years, Mpox has resurged significantly, spreading across non‐endemic regions.

In 2022, Mpox spread unexpectedly across multiple countries outside Africa, leading to thousands of confirmed cases in Europe, the Americas, and Asia. This widespread outbreak highlighted the virus's potential to emerge as a global health threat.^[^ ^20^, ^21^, ^22^, ^23^, ^24^, ^25^ ^]^

Unlike previous outbreaks, which were largely contained within rural African regions, the 2022 resurgence involved significant person‐to‐person transmission, particularly among social networks, indicating the risk of rapid spread in densely populated areas.^[^ ^26^ ^]^ By 2024, reports indicated that over 100,000 individuals worldwide have contracted Mpox.^[^ ^27^ ^]^

This resurgence has raised concerns about global preparedness for emerging zoonotic diseases. Although the mortality rate of Mpox is significantly lower than that of smallpox, severe complications can arise, especially in immunocompromised individuals and children.

The absence of a specific antiviral treatment for Mpox and reliance on smallpox vaccines has created a significant therapeutic gap, prompting an urgent need for novel antiviral strategies. Mpox's resurgence reflects a broader challenge: the potential for viruses once limited to certain regions to spread globally due to factors like increased human mobility, ecological disruption, and climate change. This highlights the need for innovative therapeutic approaches, like the development of nanohybridized niclosamide, to prevent localized outbreaks from escalating into global health crises.^[^ ^28^ ^]^ Current challenges in Mpox treatment stem from the lack of virus‐specific antiviral drugs. While options such as Tecovirimat,^[^ ^5^, ^6^, ^29^, ^30^, ^31^ ^]^ Cidofovir,^[^ ^7^, ^32^, ^33^, ^34^, ^35^, ^36^ ^]^ and Brincidofovir^[^ ^8^, ^32^ ^]^ have been repurposed from smallpox treatment, the human trial data verifying their efficacy against Mpox is limited. Vaccine limitations also pose significant issues; while smallpox vaccines like JYNNEOS^[^ ^37^, ^38^, ^39^ ^]^ and ACAM2000^[^ ^40^ ^]^ offer cross‐protection, concerns remain regarding their availability, safety (especially for immunocompromised individuals), and effectiveness in halting transmission during outbreaks. The global response has been further hindered by inadequate vaccine supply, delayed rollouts in non‐endemic regions, and the complexities of managing both zoonotic and human‐to‐human transmission, highlighting the necessity for improved therapeutic and preventive strategies.

Niclosamide is a well‐established anti‐parasitic drug, that has been used for over 50 years, primarily to treat tapeworm infections.^[^ ^41^, ^42^, ^43^ ^]^ Approved by the World Health Organization (WHO) and included in its list of essential medicines, niclosamide disrupts the energy metabolism of parasites, making it highly effective for gastrointestinal infestations.^[^ ^42^, ^44^, ^45^ ^]^ Recently, it has garnered attention for its broad‐spectrum antiviral BSA) properties, demonstrating potential against viruses such as corona, Zika, dengue, and poxv ones due to its ability to inhibit viral replication and modulate immune responses.^[^ ^46^, ^47^ ^]^ Its established safety profile, affordability, and production infrastructure make it a strong candidate for repurposing as an antiviral, including its potential application against Mpox. However, challenges like poor bioavailability and limited solubility have prompted research into innovative formulations, including nanohybridization, to improve its antiviral efficacy.^[^ ^42^ ^]^

Niclosamide exhibits distinct yet interrelated mechanisms as an antiviral and anthelmintic agent, reflecting its versatility in targeting diverse biological processes. As an anthelmintic, niclosamide disrupts mitochondrial oxidative phosphorylation in parasites, inhibiting ATP production and energy metabolism, while causing membrane depolarization, leading to ATP depletion and ultimately parasite death.^[^ ^48^ ^]^ In contrast, niclosamide's antiviral activity stems from its targeting of host‐virus interactions by inhibiting endosomal acidification, essential for viral entry, and suppressing viral replication by modulating host pathways such as Wnt/β‐catenin and NF‐κB signaling.^[^ ^49^ ^]^ These dual mechanisms underscore niclosamide's multifunctional properties, with its antiviral effects broadening its therapeutic potential beyond parasitic infections to addressing viral threats like SARS‐CoV‐2 and Mpox.

## 2 Need for Innovative Approaches

Emerging viral threats like SARS‐CoV‐2, Mpox, and dengue continue to challenge global health systems, there is an urgent need for innovative therapeutic strategies to combat them. Traditional antiviral treatments have often been limited by narrow targeting and poor bioavailability, restricting their effectiveness in rapidly evolving pandemic situations. Broad‐spectrum antivirals (BSAs), which can act on multiple viral families, are crucial for faster, more adaptable responses. New approaches, such as nano‐engineered drug formulations like NIC‐MgO‐HPMC,^[^ ^42^ ^]^ represent a breakthrough in addressing these limitations. Such innovative delivery systems enhance solubility, bioavailability, and targeted action, offering hope for more effective treatment options, particularly for post‐viral conditions like COVID‐19.

### 2.1 Definition and Key Principles of Nanohybridized Drugs

Nanohybridized drugs refer to pharmaceutical formulations that integrate nanoscale materials with active therapeutic agents to enhance their performance and bioavailability.^[^ ^50^ ^]^ These formulations combine the properties of both nanomaterials and conventional drugs to create a synergistic effect, improving drug solubility, stability, and targeted delivery. The key principles of nanohybridized drugs include:

1. Enhanced Bioavailability: Nanoparticles increase the solubility of poorly water‐soluble drugs, leading to better absorption and bioavailability.
2. Targeted Delivery: Nanocarriers can be engineered to deliver drugs specifically to diseased tissues, minimizing off‐target effects and reducing systemic toxicity.
3. 3Sustained Release: Nanomaterials can control the release of the drug over time, ensuring prolonged therapeutic effects with fewer doses.
4. Improved Stability: The incorporation of nanomaterials helps stabilize the drug in biological environments, protecting it from premature degradation.
5. Reduced Toxicity: By delivering drugs in a more controlled manner, nanohybridized systems can reduce the side effects associated with conventional dosing.

These principles enable the development of more effective treatments, especially for complex diseases like cancer, viral infections, and chronic conditions.

Niclosamide acts as a BSA due to its multifaceted mechanisms that target fundamental biomolecular processes shared across diverse viral families (Figure 2). It effectively inhibits endosomal acidification, a crucial step for viral entry and uncoating, applicable to enveloped viruses like coronaviruses, flaviviruses (Zika, dengue), as well as large DNA viruses like poxviruses, which possess both a lipid envelope and a complex protein coat. Coronaviruses and flaviviruses rely on their lipid bilayer envelope and endosomal acidification for processes such as spike protein activation (coronaviruses) and genome release (flaviviruses), both of which are disrupted by niclosamide. Poxviruses (e.g., Mpox) rely on host cellular pathways for replication, and niclosamide inhibits their replication by targeting pathways such as STAT‐3.^[^ ^51^ ^]^ Furthermore, niclosamide modulates key host cellular pathways, such as the Wnt/β‐catenin and NF‐κB signaling pathways, which are commonly hijacked by viruses to enhance replication and evade immune responses. Its suppression of autophagy further highlights its versatility by restricting viral replication in various cellular environments.^[^ ^52^, ^53^ ^]^ From lipid‐enveloped viruses like coronaviruses and flaviviruses to structurally complex viruses like poxviruses, niclosamide targets their shared dependence on host‐cell mechanisms, making it a promising antiviral candidate against a wide array of pathogens.

**Figure 1:**
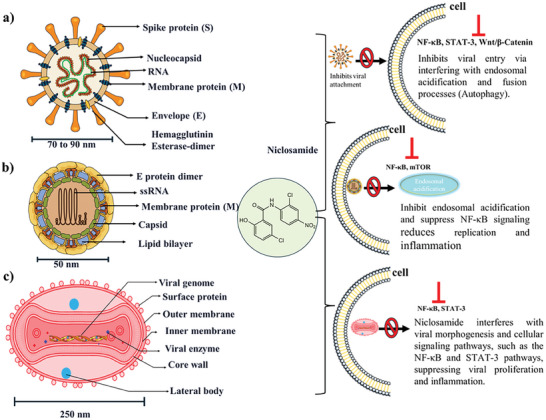
Niclosamide's action on a) coronavirus; b) flavivurus and c) poxvirus by different action mechanisms; Coronaviruses: Niclosamide prevents viral entry by interfering with endosomal acidification and fusion processes. It also suppresses viral replication by inhibiting autophagy pathways critical for coronavirus proliferation; Zika and Dengue Viruses: Both belong to the flavivirus family, relying on endocytosis and subsequent acidification for entry. Niclosamide's ability to inhibit endosomal acidification and suppress NF‐κB signaling reduces replication and inflammation; Poxviruses (e.g., Mpox): Unlike coronaviruses and flaviviruses, poxviruses replicate in the cytoplasm. Niclosamide interferes with viral morphogenesis and cellular signaling pathways, such as the NF‐κB, suppressing viral proliferation and inflammation.

Niclosamide shows promise against poxviruses due to its ability to inhibit viral replication and block critical cellular pathways exploited by viruses.^[^ ^54^ ^]^ Preclinical studies have demonstrated its efficacy against various viral families (**Figure** **1**), including orthopoxviruses, which are closely related to Mpox.^[^ ^42^ ^]^ Repurposing niclosamide for Mpox is supported by its BSA activity, favorable safety profile, and a well‐documented history of use as an FDA‐approved drug. Given the limited antiviral options for Mpox and the growing concern over outbreaks, repurposing niclosamide offers a promising, cost‐effective strategy to address both therapeutic gaps and challenges in managing Mpox infections.

### 2.2 Nanohybridization: Enhancing Drug Delivery

**Figure 2:**
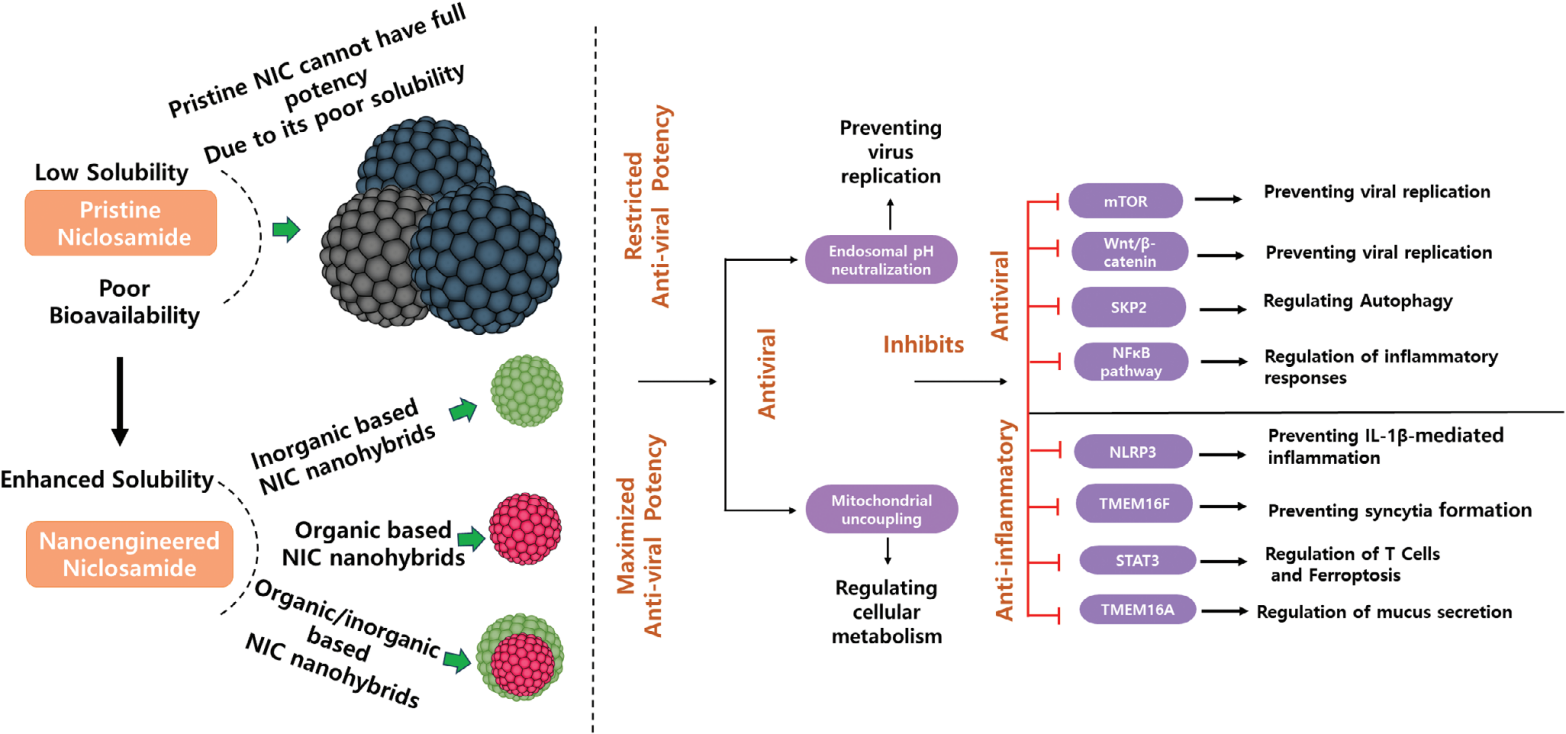
Nanoengineered niclosamide and its potential mechanisms toward Mpox and related infectious diseases.

Nanohybridized drugs are advanced pharmaceutical formulations that combine therapeutic agents with nanoscale materials to enhance their efficacy.^[^ ^55^ ^]^ By improving drug solubility and stability, these nanoformulations increase bioavailability and allow for more efficient absorption in the body (Section 2). A key advantage is targeted delivery, which directs the drug specifically to diseased tissues, reducing side effects and systemic toxicity. Additionally, nanohybridized systems allow a  controlled and sustained release, ensuring prolonged therapeutic effects with fewer doses. These innovations make nanohybridized drugs particularly valuable for treating complex conditions, including cancer, viral infections, and chronic diseases, where traditional treatments may fall short (**Figure** 2).

#### 2.2.1 Why Nanohybridized Niclosamide?

Nanohybridized niclosamide addresses the drug's historical limitations, particularly its poor solubility and low bioavailability.^[^ ^56^, ^57^ ^]^ While niclosamide is effective as an antiparasitic and shows potential in antiviral applications, these pharmacological challenges have restricted its systemic therapeutic use. By leveraging nanotechnology, niclosamide can be hybridized with materials such as magnesium oxide (MgO) and hydroxypropyl methylcellulose (HPMC), enhancing its solubility, stability, and targeted delivery.^[^ ^58^ ^]^ This improves absorption, accelerates the onset of action, and enables controlled release, making it a more viable candidate for treating viral infections like COVID‐19,^[^ ^59^ ^]^ Long COVID,^[^ ^60^ ^]^ and emerging diseases like Mpox.^[^ ^61^ ^]^

### 2.3 Previous Success in Other Viral Models

Nanotechnology has been increasingly utilized to enhance antiviral treatments by improving drug delivery, bioavailability, and efficacy while minimizing side effects.^[^ ^62^, ^63^, ^64^, ^65^, ^66^, ^67^ ^]^

**Table** **2** summarizes key applications of nanotechnology in antiviral treatments, including nanomaterials used, specific antiviral agents, and their benefits.

**Table 2:** Nanohybridization technology‐based antivirals in the market.

Nanotechnology Used	Materials involved	Antiviral Treatment	Mechanism/Advantage	Ref.
Organic based Nanotechnology	Liposome‐Based Antiviral Delivery	Acyclovir (Herpes Simplex Virus)	Enhanced stability, sustained release, higher bioavailability, and reduced side effects	[68]
		Amphotericin B (HIV, CMV)	Reduced toxicity (especially renal), improved safety profile	[69]
	Gold Nanoparticles	Oseltamivir (Tamiflu, Influenza)	Enhanced bioavailability and degradation resistance	[70]
	Polymeric Nanoparticles	Tenofovir (HIV)	Enhanced absorption, sustained release, reduced frequency of dosing, and fewer side effects	[71]
		Saquinavir (HIV)	Improved solubility, targeted delivery, reduced drug resistance	[72]
	Nanofibers	Acyclovir/Valacyclovir (HSV‐2, Genital Herpes)	Localized sustained release, improved retention, reduced systemic absorption.	[73]
	Hydrogels	HSV‐2 (Herpes Simplex Virus)	Enhanced topical drug release, better skin/mucosal penetration	[74]
	Virus‐Like Particles (VLPs)	HPV, Hepatitis B Vaccines (e.g., Gardasil, Cervarix)	Strong immune response, non‐infectious, reduced risk	[75]
	Nanoemulsions	Flu vaccines	Enhanced immune response, smaller dose required	[76]
	Nanoemulsions	Acyclovir, Penciclovir (Topical HSV treatment)	Enhanced skin penetration, prolonged drug effect, improved stability and bioavailability	[77]
Inorganic and Carbon allotrope‐based nanotechnology	Carbon Nanotubes (CNTs)	Ribavirin (RSV, Hepatitis C)	Improved targeting to infected cells, reduced toxicity	[78]
	Silver Nanoparticles	HIV, Hepatitis B Virus (HBV), Respiratory Syncytial Virus (RSV)	Broad‐spectrum antiviral activity, inhibition of viral entry, and replication prevention	[79, 80, 81]
	Selenium nanoparticles	with amantadine (H1N1 influenza virus)	Inhibition of ‐induced apoptosis by functionalized selenium nanoparticles through ROS‐mediated AKT signaling pathways	[82]
Inorganic/organic hybrids	MgO and HPMC have been used to Nanohybridized niclosamide	SARS‐CoV‐2	Enhanced solubility, thereby improving PK, and efficacy in pre‐clinical and clinical studies (phase‐3 application is in progress)	[59]

### 2.4 Mechanism of Action of nanohybridized Niclosamide Against Mpox

In‐vitro studies suggest that pristine niclosamide holds potential as a therapeutic candidate against Mpox by inhibiting viral entry and replication^[^ ^54^ ^]^ (**Table** **3**). However, due to its hydrophobic nature, traditional niclosamide formulations have limited efficacy in antiviral applications.^[^ ^54^, ^83^ ^]^

**Table 3:** Mechanistic action of niclosamide against various viral species.

Mechanism	Effect on Virus	Targeted Viruses	Ref.
Inhibition of Mitochondrial Function	Disrupts energy production, inhibiting viral replication	SARS‐CoV‐2 Zika Dengue Influenza	[93, 94, 95]
Autophagy Inhibition	Reduces availability of cellular resources for replication	SARS‐CoV‐2 Zika Dengue	[96, 97, 98]
Inhibition of mTOR Pathway	Decreases viral protein synthesis	SARS‐CoV‐2, other RNA viruses such as Epstein‐Barr virus	[47, 48, 49, 50, 51, 52, 53, 54, 55, 56, 57, 58, 59, 60, 61, 62, 63, 64, 65, 66, 67, 68, 69, 70, 71, 72, 73, 74, 75, 76, 77, 78, 79, 80, 81, 82, 83, 84, 85, 86, 87, 88]
Prevention of Endosomal Acidification	Blocks viral uncoating and genome release	SARS‐CoV‐2, Influenza, Zika, Dengue	[99, 100, 101]
Blocking ACE2‐Spike Protein Interaction	Prevents viral entry into host cells	SARS‐CoV‐2 (COVID‐19)	[102, 103]
Inhibition of PIKfyve and TMEM16	Disrupts endosomal fusion and viral entry	SARS‐CoV‐2, Zika, other enveloped viruses	[104, 105]

Nanohybridized niclosamide addresses this by enhancing solubility and bioavailability, providing superior inhibition of viral processes, including entry, replication, and dissemination. This approach maximizes niclosamide's therapeutic potential as an antiviral treatment for Mpox. While pristine niclosamide has demonstrated potential antiviral effects in in vitro studies, its efficacy can be significantly enhanced through nanohybridization techniques. This approach maximizes niclosamide's potency through various mechanisms, as illustrated in Figure **2**.

Nanohybridized niclosamide interacts with oncoproteins expressed by virus‐infected cells by binding to these proteins, disrupting their function and hindering their role in promoting viral replication.^[^ ^84^ ^]^ Oncoproteins, induced by poxviruses including Mpox, play a critical role in cellular transformation and the progression of viral replication.^[^ ^85^ ^]^ By targeting these oncoproteins, nanohybridized niclosamide effectively interferes with viral replication at the cellular level. Moreover, nanohybridized niclosamide has the potential to inhibit viral release from infected cells. Through binding to intracellular viral particles or viral envelope proteins, it prevents the packaging and release of new viral progeny, thus reducing the spread of the virus. This dual mechanism—disrupting oncoprotein function and inhibiting viral release—makes nanohybridized niclosamide a potent therapeutic agent against viral infections, including Mpox.

#### 2.4.1 Inhibition of Viral Entry

Nanohybridized niclosamide can block the entry of the Mpox virus into host cells by targeting viral envelope proteins, preventing the virus from binding to and fusing with the host cell membrane. The nano formulation enhances cellular uptake, leading to more effective inhibition at the initial stage of infection.^[^ ^86^, ^87^ ^]^

#### 2.4.2 Inhibition of Viral Replication

Once inside the host cell, nanohybridized niclosamide can inhibit viral replication by disrupting key viral enzymes and proteins required for viral RNA synthesis. The nanoscale delivery system ensures sustained release of the drug, maintaining effective concentrations at the site of infection for a longer duration. The major pathways associated with this mechanism include the inhibition of mTOR^[^ ^88^ ^]^ and Wnt/β‐catenin,^[^ ^89^ ^]^ thereby reducing viral replication.

#### 2.4.3 Disruption of Cellular Signaling

Niclosamide interferes with cellular pathways like Wnt/β‐catenin and NF‐κB signaling, which Mpox uses to promote viral replication and evade the immune system. Nanohybridization improves bioavailability and solubility, allowing better intracellular targeting and enhanced antiviral action.^[^ ^90^ ^]^

#### 2.4.4 Reduction of Inflammatory Responses

Nanohybridized niclosamide reduces the excessive inflammatory response triggered by Mpox infection, which is a major cause of severe symptoms. The nanosystem localizes the drug to the infected tissues, minimizing systemic toxicity while effectively reducing inflammation. The major signaling pathways associated with niclosamide's reduction of inflammatory responses include: NF‐κB Pathway,^[^ ^90^ ^]^ wherein niclosamide inhibits NF‐κB activation, a key transcription factor responsible for regulating inflammatory cytokines like TNF‐α, IL‐1β, and IL‐6; STAT3 Pathway, which suppresses the STAT3 signaling,^[^ ^91^ ^]^ reducing the production of pro‐inflammatory cytokines and promoting anti‐inflammatory effects; and NLRP3 Inflammasome,^[^ ^92^ ^]^ wherein niclosamide inhibits the activation of the NLRP3 inflammasome, a crucial component of the innate immune response, thus reducing IL‐1β‐mediated inflammation.

By combining these antiviral and anti‐inflammatory mechanisms with enhanced drug delivery, nanohybridized niclosamide shows promise in combating Mpox more effectively than traditional formulations.

### 2.5 Preclinical and Clinical Evidence

#### 2.5.1 In Vitro

Laboratory studies exploring the antiviral efficacy of nanohybridized niclosamide against Mpox or related viruses, such as smallpox or vaccinia, are still in the early stages, but results from other similar viral models are promising. For example:

- Nanoparticle‐based niclosamide formulations have shown enhanced antiviral effects against various enveloped viruses by improving drug delivery to infected cells, ensuring sustained release, and increasing bioavailability.^[^ ^106^ ^]^
- For vaccinia poxvirus, viral production has been shown to require elevated levels of ATP.^[^ ^107^ ^]^ If a similar mechanism applies to coronaviruses, niclosamide, by disrupting mitochondrial function and depleting cellular ATP, could exert an even stronger inhibitory effect. This suggests that niclosamide's antiviral mechanism may not only target viral replication but also interfere with the energy metabolism critical for virus production, enhancing its antiviral efficacy against both poxviruses like vaccinia and other viruses such as coronaviruses.
- Nanoliposome formulations^[^ ^93^ ^]^ have demonstrated better uptake in cells and longer‐lasting effects, which could boost niclosamide's ability to inhibit viral entry and replication in poxviruses like Mpox.

Although specific results on Mpox or smallpox are limited, the combination of niclosamide with nanotechnology shows potential to improve its effectiveness against poxvirus infections by enhancing delivery and targeting key viral processes.

Recently such an advantageous nanohybrid, known as NIC‐MgO‐HPMC (nanohybridized niclosamide with magnesium oxide and hydroxypropyl methylcellulose) was reported in antiviral therapy with an ability to overcome key challenges associated with conventional niclosamide which has very low efficacy.^[^ ^59^ ^]^ The incorporation of magnesium oxide (MgO) and hydroxypropyl methylcellulose (HPMC) into the nanohybridized formulation of niclosamide enhances its stability, bioavailability, and efficiency as an oral drug for COVID‐19 treatment. MgO prevents the weakly acidic niclosamide from crystallizing in the stomach, thereby improving its solubility for better absorption in the intestine. HPMC functions as a polymeric carrier, forming a gel‐like matrix that enables controlled and sustained drug release, ensuring therapeutic concentrations over time. This combination prevents niclosamide from forming an unabsorbable crystallized form and helps it reach the small intestine, where absorption is optimized. To further protect the drug and enhance targeting, the formulation can include enteric coatings to bypass the stomach and release the drug in neutral pH conditions, as well as functionalized ligands or stimuli‐responsive properties to ensure site‐specific delivery to virus‐infected tissues, such as the lungs. Together, these features ensure that the nanohybridized niclosamide is stable, effectively delivered, and optimally targeted, making it a promising oral therapy for COVID‐19.

#### 2.5.2 In Vivo

As of now, there are no widely reported studies specifically investigating nanohybridized niclosamide formulations in animal models for Mpox. However, based on research in related fields, animal model studies using nanoparticle‐based drug delivery systems for other viral infections show promising potential for enhanced efficacy.

Key insights from animal research in related areas include:

- Enhanced Bioavailability: Nanohybridized formulations in animal studies have shown increased bioavailability of niclosamide, allowing it to remain in the bloodstream and reach infected tissues more effectively.^[^ ^108^, ^109^ ^]^
- Reduced Viral Load: In studies of other viral infections, nanoformulations of antiviral drugs, including niclosamide, have resulted in a significant reduction of viral load in tissues and improved survival rates in animal models.^[^ ^110^, ^111^ ^]^
- Targeted Delivery: Nanocarriers have been shown to deliver drugs more efficiently to viral reservoirs, reducing off‐target effects and improving overall outcomes in animal models for similar enveloped viruses.

While no specific data is currently available for Mpox animal models, the benefits seen in other models suggest that nanohybridized niclosamide could lead to enhanced treatment outcomes, including faster viral clearance and better survival in poxvirus‐related infections (**Table** **4**).

**Table 4:** Detailed mechanism and effect on Poxviruses (e.g., Mpox).

Inhibition of Endosomal Acidification	Blocks viral uncoating and genome release into the cytoplasm, preventing infection at an early stage.	Ref.
Inhibition of Membrane Fusion (via TMEM16)	Prevents viral entry by blocking fusion between the viral envelope and host cell membrane.	[112]
Disruption of Viral Morphogenesis	Interferes with viral assembly, preventing the formation of mature viral particles.	[53]
Inhibition of mTOR Pathway	Reduces viral protein synthesis required for replication.	[113]
Autophagy Induction	Limits cellular resources available for viral replication and assembly.	[61]
Inhibition of PIKfyve and TMEM16 Proteins	Disrupts cellular trafficking and viral release from infected cells.	[114]
Inhibition of NF‐κB Signaling Inhibition of NF‐κB Signaling	Counteracts viral immune evasion and enhances host immune defense.	[114]
Anti‐inflammatory Effects	Reduces excessive inflammation caused by viral infection.	[115]

### 2.6 Ongoing or Proposed Clinical Trials of Niclosamide (Nanohybridized or Non‐Hybridized) for Viral Infections, Including Mpox

Several clinical trials are ongoing or completed, investigating niclosamide as a treatment for COVID‐19, based on its BSA properties. These trials focus on its ability to inhibit viral replication and improve outcomes in mild to moderate COVID‐19 cases. Trials are testing various formulations though nanohybridized forms are still under early exploration.

Due to niclosamide's low solubility and bioavailability, clinical trials using pristine niclosamide as an oral formulation have not been successful. A randomized, placebo‐controlled clinical trial with pristine niclosamide was previously conducted in patients with mild to moderate COVID‐19.^[^ ^116^ ^]^ In this trial, 73 participants were enrolled, with 36 randomized to the niclosamide group and 37 to the placebo group. The niclosamide group received 2g of niclosamide orally once a day for 7 days, while the placebo group received an identically labeled placebo on the same dosing schedule.

Although niclosamide was well tolerated, the results showed that there was no significant difference in the oropharyngeal clearance of SARS‐CoV‐2 between the niclosamide and placebo groups, leading to an unsuccessful outcome of the trial.

The issue of niclosamide's low bioavailability was addressed through the application of nanohybrid technology, resulting in the development of CP‐COV03 (NIC‐MgO‐HPMC: niclosamide engineered with magnesium oxide and hydroxypropyl methylcellulose), which enabled the effective use of niclosamide as an antiviral agent.^[^ ^59^ ^]^

The antiviral efficacy of CP‐COV03 was demonstrated in a Syrian hamster model, where the CP‐COV03‐treated group showed a reduced rate of SARS‐CoV‐2 replication in lung tissue. Furthermore, necropsy findings revealed reduced total lung lesions in CP‐COV03‐treated hamsters, and histological analysis showed significantly lower lung injury scores in the CP‐COV03‐treated group compared to the control group. Additionally, CP‐COV03 demonstrated safety and improved bioavailability in a Phase 1 clinical trial, further supporting its potential as an effective antiviral therapy.

While several clinical trials of niclosamide have focused on its efficacy against SARS‐CoV‐2, its potential antiviral activity against poxviruses, including Mpox, has also been explored in preclinical studies. Niclosamide has demonstrated broad‐spectrum antiviral properties, including the ability to inhibit viral replication in poxviruses by targeting intracellular pathways such as autophagy, endosomal acidification, and viral assembly. Studies using in silico host‐pathogen interaction (HPI) network models to investigate niclosamide as an antiviral agent against orthopoxviruses have shown that niclosamide can disrupt viral replication by modulating host cellular mechanisms critical for the viral life cycle.^[^ ^61^ ^]^ Although specific clinical trials for niclosamide against poxviruses are limited, preclinical evidence supports its potential application as an antiviral therapy. Expanding the clinical focus to include its use against poxviruses, either alone or in combination with other therapeutics, could provide valuable insights into its efficacy for treating viral infections like Mpox. These findings reinforce the rationale for advancing nanohybridized niclosamide as a promising therapeutic strategy for poxvirus‐related diseases.

#### 2.6.1 Niclosamide for Other Viral Infections

While there are no large‐scale trials for Mpox specifically, there is growing interest in testing niclosamide against poxviruses due to its antiviral activity in vitro. Some trials focus on its use against other viral diseases, such as Hepatitis C and SARS‐CoV‐2, which could lay the groundwork for trials in poxviruses.

### 2.7 Safety, Dosing, and Efficacy Data

#### 2.7.1 Safety

Niclosamide has a well‐established safety profile as an anti‐parasitic drug^[^ ^46^, ^117^, ^118^ ^]^ with few adverse effects when taken orally for tapeworm infections. In COVID‐19 clinical trials, niclosamide has generally been well tolerated, with side effects including mild gastrointestinal symptoms (nausea, diarrhea) and rare cases of allergic reactions. While the nanohybridized form is still under investigation, early studies suggest that nanocarrier systems can reduce systemic toxicity by targeting drug delivery more specifically to infected cells.

While niclosamide is an FDA‐approved drug with a well‐established safety profile, the introduction of nanohybridized niclosamide requires a comprehensive biosafety evaluation of the designed materials. Although there are few studies related to the safety of nanohybridized niclosamide,^[^ ^59^ ^]^ it is critical to ensure the safety of these nanohybrids, by addressing several key factors including cytotoxicity, immunogenicity, organ toxicity, and potential genotoxicity. In vitro, cytotoxicity can be evaluated using assays such as MTT or LDH to assess cell viability and membrane integrity. In vivo evaluations, using animal models, are essential to investigate organ‐specific toxicity, biodistribution, and long‐term effects of nanohybridized niclosamide. Additionally, the biodegradability and biocompatibility of the nanohybrid materials should be tested to ensure that they do not accumulate in tissues or provoke adverse immune responses. By conducting a series of rigorous biosafety tests, one can establish a well‐defined safety profile for nanohybridized niclosamide, ensuring its safe application in antiviral therapy.

#### 2.7.2 Dosing

Traditional niclosamide's low oral bioavailability has led to the development of alternative formulations, such as inhalable or intranasal forms, to improve absorption. In COVID‐19 trials, oral doses typically range from 1‐2g daily. For nanohybridized versions, lower doses are expected due to improved delivery and retention, though precise clinical data are still emerging.

#### 2.7.3 Efficacy

In‐vitro studies demonstrate that niclosamide has potent antiviral activity against a range of viruses, including SARS‐CoV‐2, Zika, Dengue, and Influenza. Early COVID‐19 trials have shown mixed results, with some studies reporting a reduction in viral load, while others suggest a limited impact on clinical outcomes. These discrepancies are likely related to the challenges of bioavailability in the non‐hybridized form.

Nanohybridized formulations have shown promising results in preclinical models for increasing antiviral efficacy by improving drug stability and targeted delivery, although clinical trial data remains limited. Ongoing clinical trials are testing niclosamide for viral infections like COVID‐19; however, none of any current trials focus on Mpox. While nanohybridized formulations are in the early stages, they show potential for improved safety and efficacy. Trials in this area are likely to emerge as interest in BSAs grows.

In addition to the potential of nano‐engineered niclosamide as an antiviral agent, it is crucial to consider other strategies, such as vaccination, to prevent the spread of Mpox. Vaccines play a vital role in controlling viral infections by stimulating the immune system to recognize and combat the virus before it can cause widespread illness. Current Mpox vaccines (**Table** **5**), such as the JYNNEOS vaccine,^[^ ^122^ ^]^ have shown effectiveness in preventing infection and reducing transmission. Combining vaccination with antiviral therapies like nano‐engineered niclosamide could provide a comprehensive strategy for managing and ultimately controlling the Mpox outbreaks.

**Table 5:** Current status of vaccines against Mpox, their mechanisms of action, and their role in preventing the spread of the virus.

Vaccine Name	Type	Mechanism of Action	Current Status	Role in Preventing Mpox Spread	Ref.
JYNNEOS (Imvamune, Imvanex)	Live, non‐replicating (Modified Vaccinia Ankara ‐ MVA)	Stimulates innate and adaptive immunity, including T‐cell activation and neutralizing antibody production.	Approved for Mpox and smallpox prevention.	Protects high‐risk individuals, reduces the severity of infection, and limits virus transmission in outbreak settings.	[119]
ACAM2000	Live, replicating (Vaccinia virus)	Induces a robust immune response through replication of the vaccinia virus, triggering antibody and T‐cell responses.	Approved but less preferred due to safety concerns.	Effective in preventing Mpox but associated with higher risks of side effects, making it suitable for specific populations.	[40]
LC16m8	Attenuated live vaccine	Similar to ACAM2000 but with reduced replication capacity, leading to lower side effects while inducing immunity.	Approved in Japan for smallpox.	Potential use in Mpox outbreaks, with a safer profile compared to traditional live vaccines.	[120]
Third‐generation vaccines	Subunit, DNA, or protein‐based platforms	Use viral proteins or genetic material to stimulate specific immune responses without using live viruses.	Under development and preclinical trials.	Expected to provide safer and more targeted immunization options with minimal adverse reactions.	[121]

This multifaceted approach, targeting both prevention and treatment, is essential for enhancing global efforts to mitigate the impact of viral infections like Mpox.

However, Vaccines alone cannot completely stop Mpox due to several limitations and challenges associated with vaccination strategies. First, limited global vaccine access and insufficient vaccine coverage in endemic regions make it difficult to achieve widespread immunity.^[^ ^123^ ^]^ Second, certain populations, such as individuals with weakened immune systems, may not respond effectively to vaccines or may face risks of adverse effects, particularly with live‐attenuated vaccines like ACAM2000. Additionally, vaccine‐induced immunity may not be immediate, as it takes time for the body to generate an adequate immune response, leaving a window of vulnerability during outbreaks.^[^ ^124^ ^]^ Variability in vaccine efficacy against different strains of the virus and the possibility of immune escape mechanisms further complicate their effectiveness as a standalone solution.

Nanohybridized niclosamide offers a complementary therapeutic strategy to address these gaps. Unlike vaccines, which primarily aim to prevent infection, nanohybridized niclosamide targets active viral infections, inhibiting viral replication, modulating host cellular pathways like autophagy and endosomal acidification, and suppressing virus release from infected cells. The nanohybrid formulation enhances drug delivery by improving the bioavailability, cellular uptake, and tissue penetration of niclosamide, enabling it to accumulate effectively at the site of infection. This approach ensures rapid antiviral activity, even after the onset of infection, and can also mitigate severe disease progression in individuals who have not been vaccinated or have compromised immunity. By combining the preventive potential of vaccines with the therapeutic efficacy of nanohybridized niclosamide, a comprehensive strategy can be achieved to control Mpox outbreaks, limit transmission, and reduce disease burden.

### 2.8 Strategic Implications for Global Health

Following the failure of the NIH‐cosponsored study PALM007, conducted in the Democratic Republic of the Congo, to meet its primary endpoint, there is currently no treatment available to curb the global spread of Mpox. In this context, nanohybridized niclosamide offers a potential solution by enhancing antiviral effectiveness through targeted and sustained delivery to infected cells (Figure 2).

As a repurposed drug already approved for the treatment of parasitic infections, niclosamide can be rapidly deployed during outbreaks, enabling a swift response to emerging viral threats. Its broad‐spectrum activity allows it to combat multiple viruses, which could help reduce hospitalizations and healthcare costs. Additionally, nanohybridization may enhance safety by minimizing side effects, making it suitable for use even in vulnerable populations.

This approach not only strengthens preparedness for poxvirus outbreaks but also promotes research into the development of more effective antiviral therapies.

Nanohybridized niclosamide has broad applicability beyond Mpox, showing promise in other viral outbreak treatments such as smallpox and emerging zoonotic viruses like Nipah and Zika. In cases where Mpox co‐infects individuals with HIV, the mortality risk increases. The antiviral activity of niclosamide against HIV suggests that Nanohybridized niclosamide could serve as a treatment option for co‐infected individuals efficacy against poxviruses also positions it as a viable therapeutic candidate for smallpox, especially in response to potential bioterrorism threats.

Additionally, the enhanced bioavailability and targeted delivery achieved through nanohybridization improve its efficacy against various viruses, including those with unique treatment challenges. As a repurposed drug, niclosamide's rapid deployment during outbreaks is essential for controlling viral spread. Overall, its demonstrated success in treating different viral infections could catalyze further research and innovations in antiviral therapies, benefiting global health by addressing a wide range of viral threats.

## 3 Conclusion 

Niclosamide holds substantial promise as a broad‐spectrum antiviral, with unique mechanisms that not only degrade viruses through autophagy but also inhibit viral entry, replication, and inflammation. However, its inherent low bioavailability has limited its clinical potential as an antiviral agent. Recent advances in nanotechnology provide solutions to these challenges, enabling niclosamide's effective use through enhanced solubility, stability, and targeted delivery mechanisms. Moreover, recent formulations with improved bioavailability through nanotechnology have shown safety in clinical trials. By incorporating niclosamide into nanostructures such as layered double hydroxides (LDHs), metal‐organic frameworks (MOFs), or polymeric nanoparticles, researchers can achieve improved pharmacokinetics and therapeutic efficacy, ensuring its viability as a tool in combating viral threats like Mpox. This approach positions niclosamide as a critical agent for addressing future pandemics, blending advanced science with accessible, scalable, and sustainable therapeutic strategies.

## 4 Prospects

### 4.1 Nanotechnology in Pandemic Preparedness

Nanohybridization technology^[^ ^125^, ^126^, ^127^, ^128^ ^]^ elevates niclosamide's role from a repurposed drug to a pivotal antiviral agent by addressing its solubility and delivery challenges. Functionalized materials like MgO nanoparticles or hydrophilic polymers such as HPMC provide effective encapsulation, ensuring that niclosamide reaches infected tissues while reducing systemic toxicity. These advancements not only improve therapeutic outcomes but also enable rapid deployment during outbreaks, ensuring global health readiness.

### 4.2 Environmentally Conscious Design

Sustainable and biodegradable nanomaterials play a key role in scaling niclosamide formulations for mass production without compromising environmental health. Eco‐friendly polymers and efficient manufacturing processes align with the global emphasis on reducing the ecological impact of pharmaceutical production. These strategies ensure that future antiviral therapies are both environmentally and economically sustainable.

### 4.3 Stimuli‐Responsive Drug Delivery

Innovative material properties, such as porosity and stimuli‐responsive capabilities, allow nanohybridized niclosamide to adapt to specific biological environments. These systems release the drug in response to environmental triggers like pH or enzymatic activity, maximizing therapeutic efficacy while minimizing wastage. This precision delivery system sets a precedent for smarter, more efficient antiviral treatments.

### 4.4 Scalable Solutions for Global Health Security

The affordability and scalability of nanohybridized niclosamide make it accessible across diverse income settings, addressing the critical need for equitable healthcare solutions. By leveraging advanced material science to develop cost‐effective formulations, niclosamide can serve as a cornerstone in global health preparedness, ensuring robust responses to viral outbreaks while enhancing resilience against future pandemics.

This integration of nanotechnology and material science underscores the potential of niclosamide to transform pandemic preparedness strategies, bridging the gap between innovation and accessibility. Future efforts must prioritize clinical trials to establish its efficacy and safety further, positioning niclosamide as a key player in global health security frameworks.

## Conflict of Interest

The authors declare no conflict of interest.

## Supporting information



Supporting Information
