# Supplementary material for: Harnessing Nanohybridized Niclosamide for Precision Mpox Therapeutics
Source: Adv Healthc Mater. 2025 Feb 23;14(14):2404818. doi: 10.1002/adhm.202404818 (PMC12118342; doi:10.1002/adhm.202404818)
Supplement: Supplementary file 1 — Supporting Information [file ADHM-14-0-s001.docx]

**Harnessing Nanohybridized Niclosamide for Precision Mpox Therapeutics**

*N. Sanoj Rejinold^1^, Geun-woo Jin ^2^ and Jin-Ho Choy ^1,3,4*^*

**N. Sanoj Rejinold^1^**

^1^Intelligent Nanohybrid Materials Laboratory (INML), Department of Chemistry, College of Science and Technology, Dankook University, Cheonan 31116, Republic of Korea

E-mail: sanojrejinold@dankook.ac.kr

**Geun-woo Jin**

^2^R&D Center, Hyundai Bioscience Co. LTD., Seoul, 03759 Republic of Korea

E-mail: geunwoo.jin@hyundaibio.com

**Jin-Ho Choy ^1,,3,4^**

^1^Intelligent Nanohybrid Materials Laboratory (INML), Department of Chemistry, College of Science and Technology, Dankook University, Cheonan 31116, Republic of Korea

^3^Division of Natural Sciences, The National Academy of Sciences, Seoul 06579, Republic of Korea

^4^Tokyo Tech World Research Hub Initiative (WRHI), Institute of Innovative Research, Institute of Science Tokyo, Yokohama 226-8503, Japan

orcid.org/0000-0002-4149-7100

*Corresponding Author **Email: jhchoy@dankook.ac.kr**

*Supporting Information*


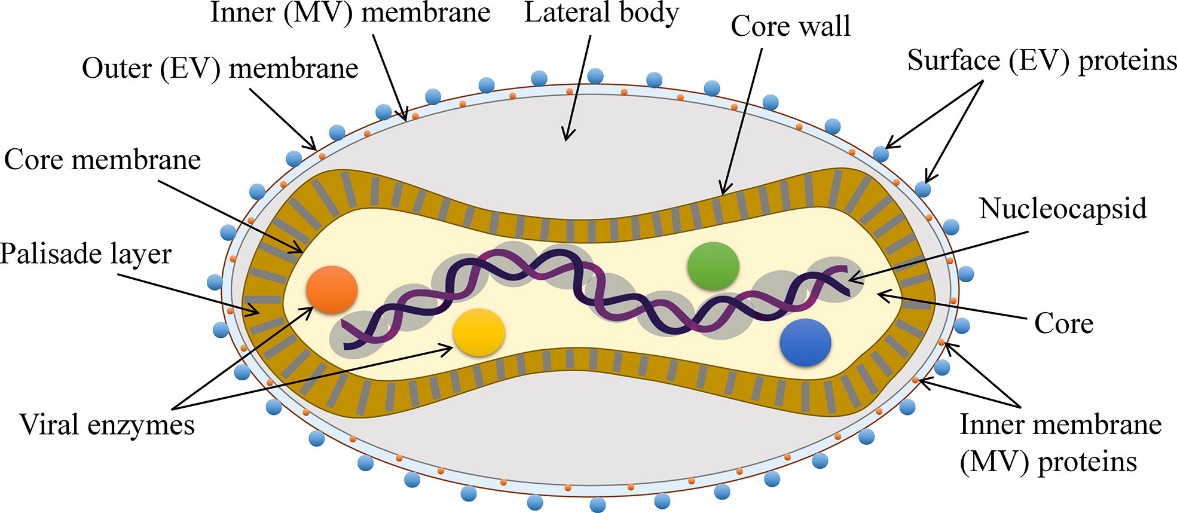


Figure S1. Structural configuration of Mpox virus. Reproduced with permission under Creative Commons Attribution License (CC BY) from [1]

[1] D. Papukashvili, N. Rcheulishvili, C. Liu, X. Wang, Y. He, P.G. Wang, Strategy of developing nucleic acid-based universal monkeypox vaccine candidates, Frontiers in Immunology 13 (2022) 1050309.
